# Supplementary material for: Live tracking of a plant pathogen outbreak reveals rapid and successive, multidecade plasmid reduction
Source: mSystems. 2024 Jan 26;9(2):e00795-23. doi: 10.1128/msystems.00795-23 (PMC10878067; doi:10.1128/msystems.00795-23)
Supplement: Supplemental figures — Fig. S1 to S3. [file msystems.00795-23-s0001.pdf]

Supplementary Materials for

Live tracking of a plant pathogen outbreak reveals rapid and successive, multidecade plasmid reduction

Veronica Roman-Reyna *et al.*

.

*Corresponding author. Email: Jacobs.1080@osu.edu

This PDF file includes:

Figs. S1 to S3

Figure legends.

Fig. S1. Chromosome genome comparisons. A) Pangenome analysis of the chromosomes represented as an upset plot. The colored bars represent the total number of proteins in each genome. The connected black dots indicate who is part of the intersection and the black bars indicated how many proteins they are sharing. B) KEGG functional annotation of Xhp chromosomes. At t-test indicated they are not significant differences across chromosomes. C) Mobile genetic elements in each Xhp chromosome annotated with MobileOG-db. D) Effector content in the Xhp chromosomes using BLASTX search. Effectors were considered present with a percentage of amino acid identity higher than 50% and coverage higher than 70%. Each ring represents a genome all aligned to dnaA sequence. Graphs and MGE annotations were made with ProkseE.

Fig. S2. Phylogenetic comparisons of proteins coded in each Xhp plasmid shared among Xanthomonadaceae. Proteins were aligned using MAFFT. Alignments were trimmed with ClipKIT and trees were constructed with IQ-TREE. Trees were rooted to best-supported outgroup (bootstrap>90).

Fig. S3. Phylogenetic tree of bacterial genomes with hits to Xhp 2012 plasmids. Yellow represents hits to p66, purple represent hits to p45, and orange represent hits to p31. If the plasmid shared 10-49% genes with the plasmid, half of the circle is gray; if the plasmids have more than 50% nucleotide identity, the circle has one color. The tree was build using Average Nucleotide Identity on the Enveomics website and visualized as a phylogenetic tree and the Ward model. +4* indicated plasmids on NCBI that had a size less than 5Kb. Question mark indicates no information in the NCBI Biosample.

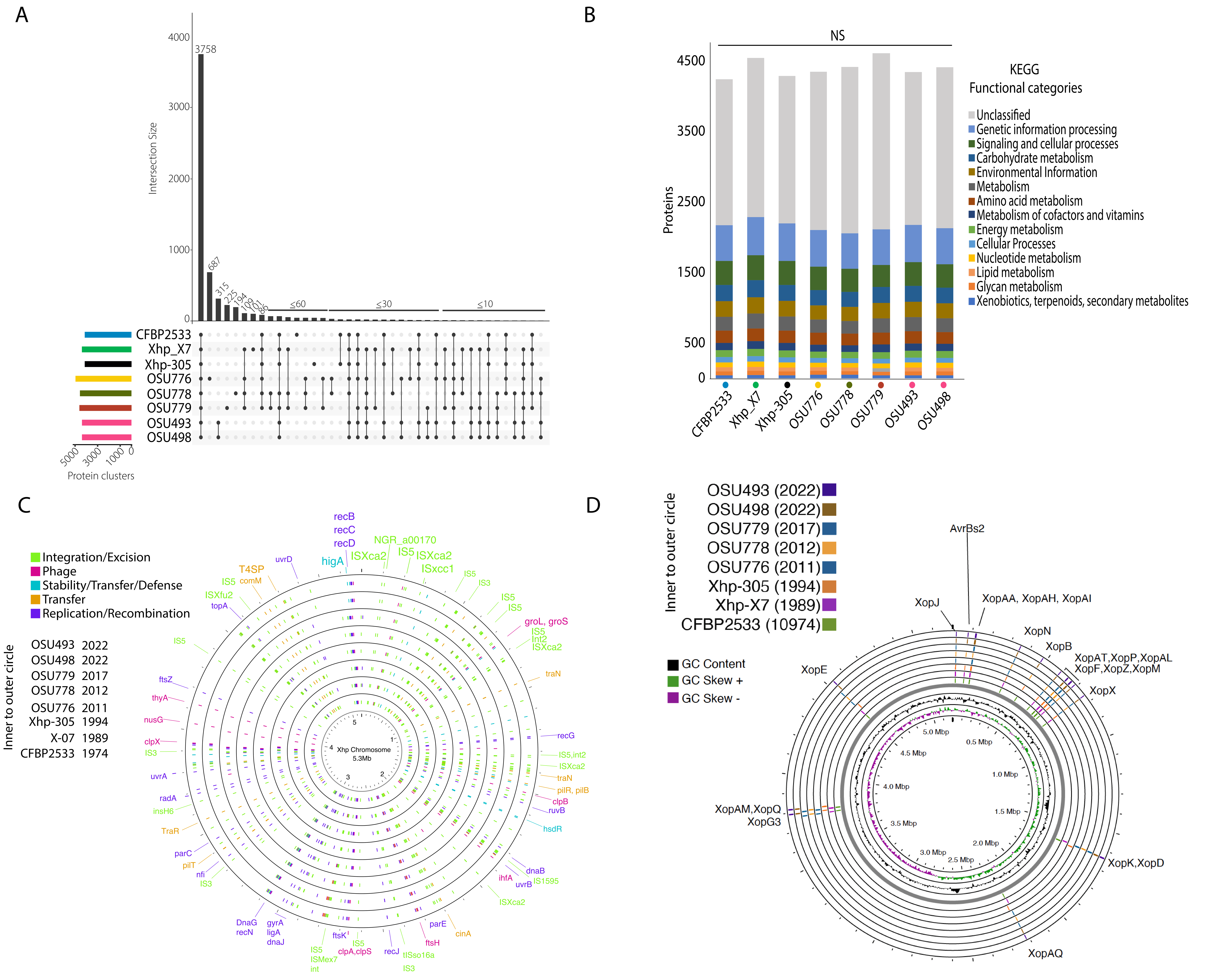

A
Intersection Size
3758
687
315
225
194
109
101
86
≤60
≤30
≤10
CFBP2533
Xhp_X7
Xhp-305
OSU776
OSU778
OSU779
OSU493
OSU498
Protein clusters
B
NS
Proteins
KEGG
Functional categories
Unclassified
Genetic information processing
Signaling and cellular processes
Carbohydrate metabolism
Environmental Information
Metabolism
Amino acid metabolism
Metabolism of cofactors and vitamins
Energy metabolism
Cellular Processes
Nucleotide metabolism
Lipid metabolism
Glycan metabolism
Xenobiotics, terpenoids, secondary metabolites
CFBP2533
Xhp_X7
Xhp-305
OSU776
OSU778
OSU779
OSU493
OSU498
C
Integration/Excision
Phage
Stability/Transfer/Defense
Transfer
Replication/Recombination
Inner to outer circle
OSU493 2022
OSU498 2022
OSU779 2017
OSU778 2012
OSU776 2011
Xhp-305 1994
X-07 1989
CFBP2533 1974
Xhp Chromosome
5.3Mb
recB
recC
recD
higA
ISXca2
NGR_a00170
IS5
ISXca2
ISXcc1
T4SP
uvrD
comM
ISXfu2
topA
groL, groS
Int2
traN
ftsZ
thyA
nusG
clpX
IS3
recG
IS5,int2
pilR, pilB
clpB
ruvB
hsdR
uvrA
radA
insH6
TraR
parC
pilT
nfi
dnaB
IS1595
ihfA
uvrB
DnaG
recN
gyrA
ligA
dnaJ
ISMex7
int
ftsK
clpA,clpS
recJ
ftsH
parE
cinA
ISSso16a
D
Inner to outer circle
OSU493 (2022)
OSU498 (2022)
OSU779 (2017)
OSU778 (2012)
OSU776 (2011)
Xhp-305 (1994)
Xhp-X7 (1989)
CFBP2533 (10974)
GC Content
GC Skew +
GC Skew -
AvrBs2
XopJ
XopAA, XopAH, XopAI
XopN
XopB
XopAT,XopP,XopAL
XopF,XopZ,XopM
XopX
XopE
XopAM,XopQ
XopG3
XopK,XopD
XopAQ
0.5 Mbp
1.0 Mbp
1.5 Mbp
2.0 Mbp
2.5 Mbp
3.0 Mbp
3.5 Mbp
4.0 Mbp
4.5 Mbp
5.0 Mbp

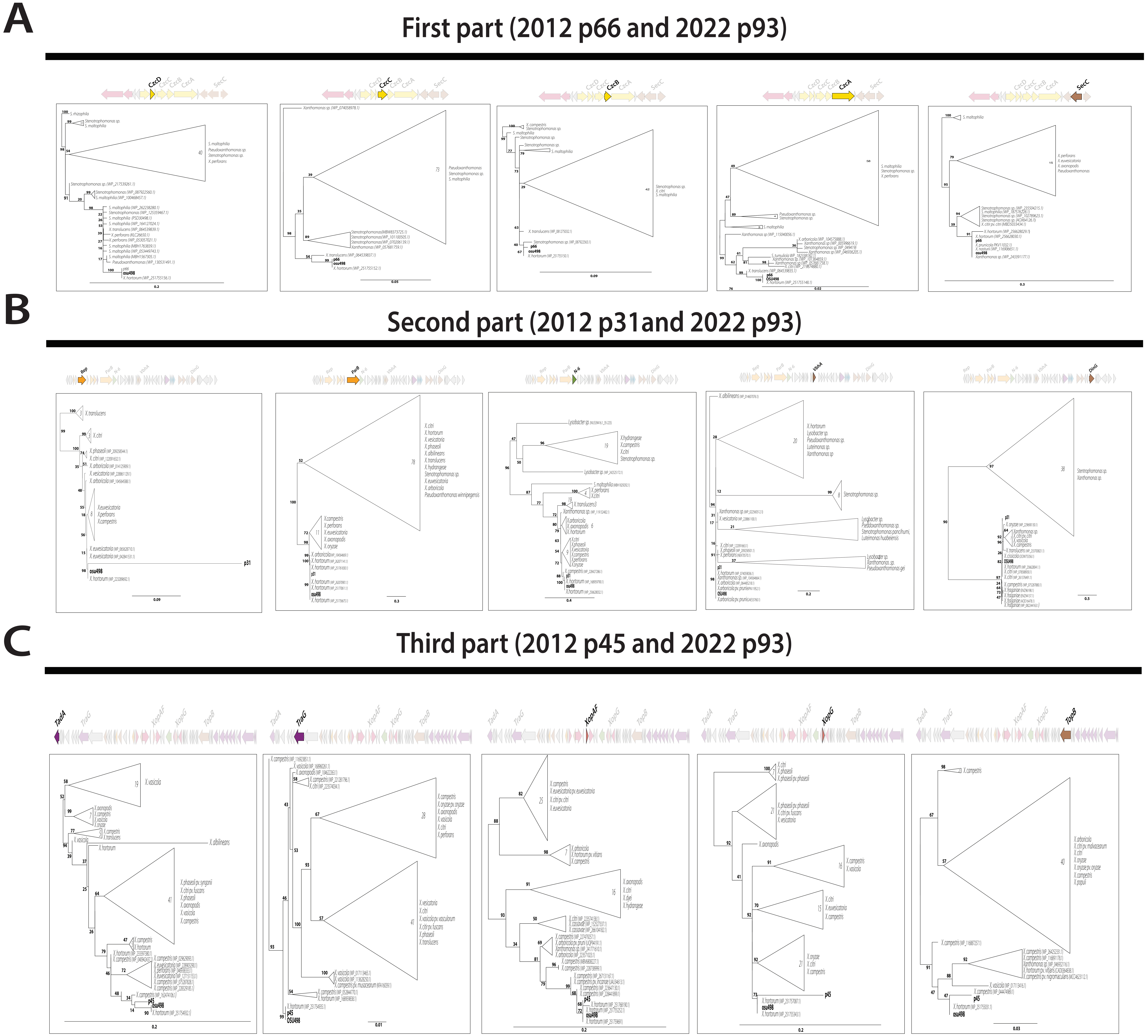
A
First part (2012 p66 and 2022 p93)
CzcD
CzcC
CzcB
CzcA
SecC
B
Second part (2012 p31and 2022 p93)
Rep
ParB
N-6
VbhA
DinG
C
Third part (2012 p45 and 2022 p93)
TadA
TraG
XopAF
XopG
TopB

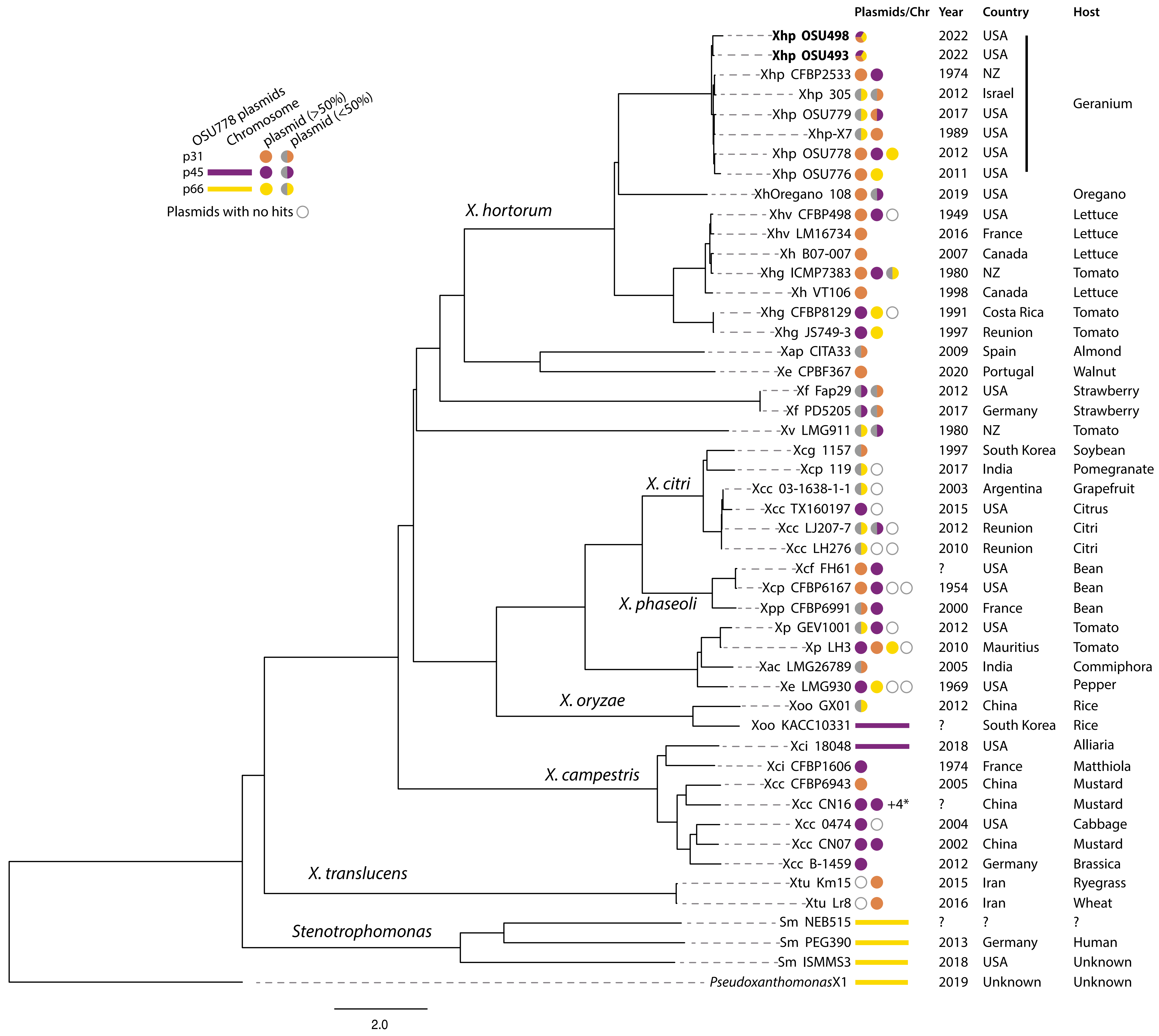
OSU778 plasmids
Chromosome
plasmid (>50%)
plasmid (<50%)
p31
p45
p66
Plasmids with no hits
Plasmids/Chr
Year
Country
Host
X. hortorum
X. citri
X. phaseoli
X. oryzae
X. campestris
X. translucens
Stenotrophomonas
Xhp OSU498 2022 USA
Xhp OSU493 2022 USA
Xhp CFBP2533 1974 NZ
Xhp 305 2012 Israel
Xhp OSU779 2017 USA
Xhp-X7 1989 USA
Xhp OSU778 2012 USA
Xhp OSU776 2011 USA
Geranium
XhOregano 108 2019 USA Oregano
Xhv CFBP498 1949 USA Lettuce
Xhv LM16734 2016 France Lettuce
Xh B07-007 2007 Canada Lettuce
Xhg ICMP7383 1980 NZ Tomato
Xh VT106 1998 Canada Lettuce
Xhg CFBP8129 1991 Costa Rica Tomato
Xhg JS749-3 1997 Reunion Tomato
Xap CITA33 2009 Spain Almond
Xe CPBF367 2020 Portugal Walnut
Xf Fap29 2012 USA Strawberry
Xf PD5205 2017 Germany Strawberry
Xv LMG911 1980 NZ Tomato
Xcg 1157 1997 South Korea Soybean
Xcp 119 2017 India Pomegranate
Xcc 03-1638-1-1 2003 Argentina Grapefruit
Xcc TX160197 2015 USA Citrus
Xcc LJ207-7 2012 Reunion Citri
Xcc LH276 2010 Reunion Citri
Xcf FH61 ? USA Bean
Xcp CFBP6167 1954 USA Bean
Xpp CFBP6991 2000 France Bean
Xp GEV1001 2012 USA Tomato
Xp LH3 2010 Mauritius Tomato
Xac LMG26789 2005 India Commiphora
Xe LMG930 1969 USA Pepper
Xoo GX01 2012 China Rice
Xoo KACC10331 ? South Korea Rice
Xci 18048 2018 USA Alliaria
Xci CFBP1606 1974 France Matthiola
Xcc CFBP6943 2005 China Mustard
Xcc CN16 +4* ? China Mustard
Xcc 0474 2004 USA Cabbage
Xcc CN07 2002 China Mustard
Xcc B-1459 2012 Germany Brassica
Xtu Km15 2015 Iran Ryegrass
Xtu Lr8 2016 Iran Wheat
Sm NEB515 ? ? ?
Sm PEG390 2013 Germany Human
Sm ISMMS3 2018 USA Unknown
PseudoxanthomonasX1 2019 Unknown Unknown
2.0
